# Randomized investigation of increased dialyzer membrane hydrophilicity on hemocompatibility and performance

**DOI:** 10.1186/s12882-024-03644-5

**Published:** 2024-07-10

**Authors:** Götz Ehlerding, Wolfgang Ries, Manuela Kempkes-Koch, Ekkehard Ziegler, Petra Ronová, Mária Krizsán, Jana Verešová, Mária Böke, Ansgar Erlenkötter, Robert Nitschel, Adam M. Zawada, James P. Kennedy, Jennifer Braun, John W. Larkin, Natalia Korolev, Thomas Lang, Bertram Ottillinger, Manuela Stauss-Grabo, Bettina Griesshaber

**Affiliations:** 1 Zentrum für Nieren-, Hochdruck- und Stoffwechselerkrankungen, 30453 Hannover, Germany; 2 Diakonissenkrankenhaus, Innere Medizin, Abtlg. Nephrologie, 24939 Flensburg, Germany; 3 PHV-Dialysezentrum Goslar, 38642 Goslar, Germany; 4 Nieren- und Gefäßzentrum Kiel, 24106 Kiel, Germany; 5 Fresenius Nephrocare Praha 9, Praha, 19061 Czechia; 6 Péterfy II. Dialízis Központ, Budapest, 1077 Hungary; 7 Fresenius Nephrocare Nymburk, Nymburk, 28802 Czechia; 8 Váci Dialízis Központ, Vác, 2600 Hungary; 9 Fresenius Medical Care Deutschland GmbH, Biosciences, VS Dialyzers, Care Enablement, 66606 St. Wendel, Germany; 10 Fresenius Medical Care Deutschland GmbH, Product Development, VS Dialyzers, Care Enablement, 66606 St. Wendel, Germany; 11 Fresenius Medical Care Deutschland GmbH, Global Biomedical Evidence Generation, Global Medical Office, 61352 Bad Homburg, Germany; 12 Fresenius Medical Care, Global Medical Office, Waltham, MA USA; 13 Ottillinger Life Sciences, 85649 Brunnthal, Germany

**Keywords:** Dialyzer, Membrane, Hydrophilicity, Hemocompatibility, Performance, Hemodiafiltration

## Abstract

**Background:**

Hemodialyzers should efficiently eliminate small and middle molecular uremic toxins and possess exceptional hemocompatibility to improve well-being of patients with end-stage kidney disease. However, performance and hemocompatibility get compromised during treatment due to adsorption of plasma proteins to the dialyzer membrane. Increased membrane hydrophilicity reduces protein adsorption to the membrane and was implemented in the novel FX CorAL dialyzer. The present randomized controlled trial compares performance and hemocompatibility profiles of the FX CorAL dialyzer to other commonly used dialyzers applied in hemodiafiltration treatments.

**Methods:**

This prospective, open, controlled, multicentric, interventional, crossover study randomized stable patients on post-dilution online hemodiafiltration (HDF) to FX CorAL 600, FX CorDiax 600 (both Fresenius Medical Care) and xevonta Hi 15 (B. Braun) each for 4 weeks. Primary outcome was β2-microglobulin removal rate (β2-m RR). Non-inferiority and superiority of FX CorAL versus comparators were tested. Secondary endpoints were RR and/or clearance of small and middle molecules, and intra- and interdialytic profiles of hemocompatibility markers, with regards to complement activation, cell activation/inflammation, platelet activation and oxidative stress. Further endpoints were patient reported outcomes (PROs) and clinical safety.

**Results:**

82 patients were included and 76 analyzed as intention-to-treat (ITT) population. FX CorAL showed the highest β2-m RR (76.28%), followed by FX CorDiax (75.69%) and xevonta (74.48%). Non-inferiority to both comparators and superiority to xevonta were statistically significant. Secondary endpoints related to middle molecules corroborated these results; performance for small molecules was comparable between dialyzers. Regarding intradialytic hemocompatibility, FX CorAL showed lower complement, white blood cell, and platelet activation. There were no differences in interdialytic hemocompatibility, PROs, or clinical safety.

**Conclusions:**

The novel FX CorAL with increased membrane hydrophilicity showed strong performance and a favorable hemocompatibility profile as compared to other commonly used dialyzers in clinical practice. Further long-term investigations should examine whether the benefits of FX CorAL will translate into improved cardiovascular and mortality endpoints.

**Trial registration:**

eMPORA III registration on 19/01/2021 at ClinicalTrials.gov (NCT04714281).

**Supplementary Information:**

The online version contains supplementary material available at 10.1186/s12882-024-03644-5.

## Introduction

During hemodialysis, one function of the dialyzer is the selective sieving of molecules [1, 2]. Middle-sized uremic toxins like β2-microglobulin (β2-m; 11.8 kDa) are in specific focus as their plasma concentration associates with patient mortality [3–8].

Hemodialysis membranes have a pro-inflammatory and pro-thrombogenic potential. Therefore, in addition to performance, the hemocompatibility of dialyzer membranes defines their benefit for patients. The most widely used synthetic polymers for membranes are polysulfone and polyethersulfone. To overcome hydrophobicity, these membranes are often blended with hydrophilic polyvinylpyrrolidone (PVP). Increased PVP content on the membrane’s blood-side surface reduces protein adsorption to the membrane, and associates with positive effects on hemocompatibility and performance [9–17].

FX CorAL 600, the investigational dialyzer in this study (Fresenius Medical Care, Bad Homburg, Germany), contains such a membrane with higher PVP content on its blood-side surface to increase membrane hydrophilicity [9, 10]. To prevent PVP oxidation and elution, it is stabilized with a small amount of α-tocopherol [9, 18].

Three earlier clinical trials evaluated FX CorAL 600 during short treatment periods [11, 19, 20]. FX CorAL showed the highest β2-m removal rates (RR) as compared to synthetic and cellulose-based comparator dialyzers. Hemocompatibility analyses showed a favorable profile for FX CorAL in terms of complement and white blood cell activation [19, 20]. In addition, FX CorAL demonstrated the lowest albumin loss up to 60 min and its sieving properties changed less over time than with comparators [11].

While these studies investigated performance and hemocompatibility during one-week treatment periods, the current study compared the performance, hemocompatibility, patients’ well-being, and safety of FX CorAL with two other high-flux dialyzers over longer periods (4 weeks per dialyzer). In addition, this study applied an extended panel of hemocompatibility markers and investigated both intra- and interdialytic profiles.

## Methods

### Trial design

eMPORA III was a multicenter, prospective, open, controlled, interventional, crossover study with randomized treatment sequences. Planning, conduct, analysis, and reporting followed Good Clinical Practice as per ISO 14155 and the Declaration of Helsinki. eMPORA III is registered at ClinicalTrials.gov (NCT04714281, registration on 19/01/2021).

### Participants

eMPORA III recruited patients on chronic online post-dilution hemodiafiltration (HDF). Eight hemodialysis centers participated, four in Germany and two each in Czechia and Hungary.

To be eligible, patients had to receive dialysis treatment 3x per week with a duration ≥ 4 h per session. Patients had to be treated with FX or FX CorDiax dialyzers (both Fresenius Medical Care) for ≥ 1 month to ensure homogenous baseline conditions. Vascular access (fistula or graft) enabling blood flow rates ≥ 300 mL/min was required. Patients with concurrent major illnesses, infections (including SARS-CoV-2 within 12 weeks prior to study), or considered clinically unstable by the investigator, with repeated failure of vascular access or with single needle treatments were not eligible, as were patients with known or suspected allergy to dialyzer and related products, with immunodeficiencies or under immunosuppression. Patients had to provide personal written informed consent.

### Interventions

This trial compared three different dialyzers: FX CorAL 600, FX CorDiax 600, and xevonta Hi 15 (Table 1). The study included three consecutive treatment periods of four weeks each, i.e., 12 sessions per dialyzer. Treatments with allocated dialyzers were started at the first mid-week visit of a treatment period. These three periods were followed by one week with the FX or FX CorDiax dialyzer type used before the study, and one follow-up visit in the subsequent week to assess overall patient’s status (Figure S1). Treatments were performed with one of the following hemodialysis machines: 5008, 5008 S or 6008 (Fresenius Medical Care). Treatment modality (i.e., post-dilution online HDF over ≥ 240 min), settings, and anticoagulation with heparin remained unchanged per patient between sessions and periods/dialyzers, unless required for medical reasons. The target blood flow rate (Qb) was ≥ 300 mL/min, and the target dialysate flow rate (Qd) ≥ 500 mL/min. The substitution flow rate (Qs) was adjusted manually to between 60 and 120 mL/min according to individual center practice.

**Table 1:** Investigated dialyzers in the present study

Dialyzer	Manufacturer	Membrane name	Membrane material	Surface [m^2^]	Sterilization method
FX CorAL 600	Fresenius Medical Care	Helixone *hydro*	Polysulfone + PVP	1.6	INLINE steam
FX CorDiax 600	Fresenius Medical Care	Helixone *plus*	Polysulfone + PVP	1.6	INLINE steam
xevonta Hi 15	B. Braun	Amembris	Polysulfone + PVP	1.5	Gamma radiation

PVP: Polyvinylpyrrolidone

### Outcome variables and laboratory methods

The primary objective of this study was to test whether FX CorAL was non-inferior and superior to the comparator hemodialyzers in removing plasma β2-m. The β2-m RR over four-hour HDF sessions was the primary endpoint (t = 0–240 min after treatment start).

Secondary performance endpoints were β2-m clearance at 60 min as well as RRs (t = 0–240 min) and clearances (t = 60 min) for myoglobin, phosphate, creatinine, and urea.

A secondary objective of this study was to compare hemocompatibility profiles between hemodialyzers, with a larger panel of hemocompatibility markers than in previous studies [9–11, 19–21]. Detailed intradialytic profiles were obtained in each trial period mid-week before the next filter switch (Table 2, Figure S1). Samples were taken pre-treatment and 15, 60 and 240 min after treatment start. Pre-treatment samples of each mid-week dialysis session were investigated for interdialytic trends.

**Table 2:** Hemocompatibility markers

Complement activation
C3a
sC5b-9
Cell activation / inflammation
Leukocyte count (total, monocytes, lymphocytes, neutrophils, eosinophils, basophils)
Polymorphonuclear (PMN) granulocyte elastase
Leukotriene B4 (LTB-4)
Soluble intercellular adhesion molecule 1 (sICAM-1)
Interleukin 6 (IL-6)
Interleukin 8 (IL-8)
High sensitivity C-reactive protein (hsCRP)
Platelet activation
Platelet count
β-thromboglobulin (β-TG)
Thromboxane B2 (TxB2)
Oxidative stress
Malondialdehyde (MDA)
Glutathione peroxidase (GSH-Px)

Methods for obtaining blood samples and laboratory methods are described in Table S1.

A further objective was the evaluation and comparison between dialyzers of symptom-related well-being through validated patient-reported outcome (PRO) measures. As symptoms may change faster than general well-being, questionnaires with short recall periods between one day and one month were chosen and administered in four-weekly intervals: before being randomized to the first, second, and third dialyzer, and before being re-assigned to the dialyzer used before the study. Questionnaires contained 25 items in native language and included the Pittsburgh Sleep Quality Index (PSQI) [22], Kidney Disease Quality of Life Short Form (KDQOL-SF) fatigue domain (questions 9a, e, g & i) [23], Pruritus Numerical Rating Scale (PNRS) [24–27], and the International Restless Legs Syndrome Study Group Rating Scale (IRLSSG; Table S2) [28–30].

### Sample size

For the primary variable β2-m RR, a non-inferiority margin of − 5% was defined, which was based on an earlier study and assumed non-inferiority at a standardized difference of 0.58 between dialyzers, and an expected standard deviation of approximately 11% [20]. As one aim of this study was to show non-inferiority of FX CorAL vs. two different comparators simultaneously, the sample size was calculated according to the Bonferroni-Holm procedure at one-sided α = 0.0125. With a statistical power of 90% for the more sensitive test and an assumed dropout rate of 15% based on earlier studies [11, 19, 20], *n* = 63 patients had to be included, corresponding to 189 dialysis periods overall. An unforeseeable additional number of dropouts was expected due to the COVID-19 pandemic, and to preserve statistical power it was planned to stop the study when 189 valid dialysis periods had been accrued, even if this required recruitment of more than 63 patients. Sample size estimation was performed with nQuery Advisor 5.0.

### Randomization

The crossover design of eMPORA III permitted six possible treatment sequences. Randomization was performed by an electronic data capture system which assigned eligible patients to an available patient number automatically (ClinDoc®, Institut Dr. Schauerte, Munich, Germany). SAS Version 9.4 (SAS Institute, Cary, NC, USA) was used for the block-wise randomization algorithm and statistical analyses.

### Statistical methods

The primary variable β2-m RR was calculated for each of the three dialyzers based on mean β2-m concentrations from the last mid-week visit in each study period. For details on calculations and statistical methods see Supplement Sect. 1.

To test non-inferiority as well as superiority of FX CorAL vs. its comparators, the primary analysis used a gatekeeping procedure to prevent inflation of the Type 1 error rate (one-sided α = 2.5%) [31]. Non-inferiority as well as superiority comparisons were based on a linear mixed model which allowed the estimation of the mean difference in the primary variable between FX CorAL and the comparators.

Secondary performance endpoints as well as hemocompatibility markers and PROs were analyzed descriptively, without testing hypotheses; p-values were not adapted for multiple testing. Calculations of RR and blood-side clearance (K_b_) are described in Supplement Sect. 1 [32, 33].

Safety events were coded in MedDRA and analyzed by preferred term, system organ class, seriousness, and relatedness to HDF or dialyzer employed at the time the event occurred.

## Results

The study was performed between February 8, 2021, and May 5, 2022. Recruitment was stopped, once 189 valid dialysis periods had been documented.

**Fig. 1:**
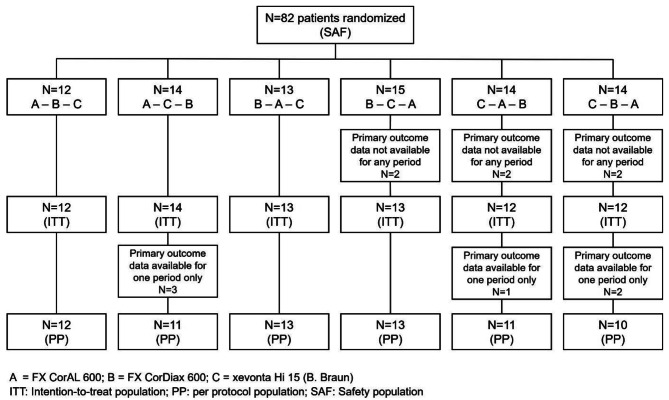
Disposition of patients

The safety population (SAF) consisted of 82 randomized patients. Primary outcome data were not available for six patients: two patients terminated the study prior to or during the first period due to personal reasons, two due to a COVID-19 infection, one due to a non-fatal serious adverse event (SAE; myocardial infarction), and one due to sudden cardiac death. Primary outcome data of further six subjects were available only for one study period: three patients terminated the study early due to personal reasons, one due to hospitalization unrelated to devices or study procedures, and two due to COVID-19. Overall, the intention-to-treat (ITT) population consisted of 76 patients and the per-protocol (PP) population of 70 patients (Fig. 1).

### Baseline data, HDF and vital signs

Table 3 shows baseline information of the study participants, Table 4 information on treatment parameters. Information on anticoagulation is presented in Table S3. There were no remarkable differences in baseline characteristics between dialyzers.

**Table 3:** Demographic and medical history data (ITT population)

	Total (*N* = 76)
Age [years]	67.0 ± 15.63
Gender [% male]	74%
BMI [kg/m^2^]	27.3 ± 5.12
Weight [kg]	
Male	81.3 ± 16.86
Female	76.2 ± 17.39
Primary renal disease* [*n* (%)]	
Hypertensive / large vessel disease	33 (43.4%)
Diabetes mellitus	26 (34.2%)
Cystic / hereditary / congenital diseases	7 (9.2%)
Glomerulonephritis	5 (6.6%)
Time on RRT [median months (range)]	39.9 (3-387)
Duration of current treatment modality [median months]	25.1
Systolic / diastolic blood pressure pre-dialysis [mmHg]	144.5 ± 20.95 / 71.6 ± 13.34
Heart rate pre-dialysis [bpm]	68.6 ± 10.18
**Concomitant diseases [MedDRA SOC / most frequent PT]**	**[% affected]**
Vascular disorders / Hypertension	96.1% / 92.1%
Metabolism and nutrition disorders / Type 2 diabetes mellitus	89.5% / 34.2%
Blood and lymphatic system disorders / Nephrogenic anemia	82.9% / 60.5%
Musculoskeletal and connective tissue disorders / CKD-mineral and bone disorder	60.5% / 30.0%
Cardiac disorders / Coronary artery disease	56.6% / 19.7%
Nervous system disorders / Carotid arteriosclerosis	51.3% / 11.8%

*More than one disease could be documented; BMI: body mass index; bpm: beats per minute; CKD: chronic kidney disease; PT: preferred term; RRT: renal replacement therapy; SOC: system organ class. Data are presented as mean ± standard deviation (SD) or number (percentage), if not indicated otherwise

**Table 4:** Treatment parameters (ITT population)

	Dialyzer		
	FX CorAL 600 (*N* = 74)	FX CorDiax 600 (*N* = 70)	xevonta Hi 15 (*N* = 74)
Blood flow rate at 60 min [mL/min]	345 ± 43.6	345 ± 41.5	345 ± 41.7
Dialysate flow rate at 60 min [mL/min]	577 ± 95.9	574 ± 97.3	578 ± 98.3
Substitution flow rate at 60 min [mL/min]	86.2 ± 9.41	85.4 ± 10.59	85.5 ± 11.02
Substitution volume at 240 min [L]	19.0 ± 2.31	18.9 ± 2.59	18.8 ± 2.75
Substitution volume at end of HDF [L]	21.0 ± 3.23	20.7 ± 3.39	20.8 ± 3.27
Ultrafiltration rate at 60 min [mL/min]	9.2 ± 4.01	9.4 ± 3.75	9.4 ± 4.17
Ultrafiltration volume at 240 min [mL]	2093.6 ± 893.65	2193.6 ± 864.32	2182.5 ± 965.65
Ultrafiltration volume at end of HDF [mL]	2313.8 ± 995.18	2372.6 ± 944.96	2414.6 ± 1093.0
Effective treatment time [min]	262 ± 21.4	259 ± 19.2	263 ± 22.9

Data are presented as mean ± standard deviation (SD)

Convection volume can be calculated as the sum of the substitution volume and the net ultrafiltration volume (i.e., the treatment-induced weight loss as calculated to estimate dry weight)

### Outcomes – primary endpoint: β2-m RR

Table 5 displays β2-m RR in the PP and ITT populations. The test for non-inferiority (PP population) of FX CorAL vs. FX CorDiax and xevonta demonstrated the non-inferiority of FX CorAL to both comparators (*p* < 0.0001 vs. each). After passing these tests, superiority of FX CorAL to the comparators was tested in the ITT population. FX CorAL was superior to xevonta (*p* < 0.0001), but not to FX CorDiax (*p* = 0.0606). Tests of fixed effects found neither sequence or period effects (*p* = 0.77) nor carry-over effects (*p* = 0.74; PP population).

**Table 5:** Primary endpoint β2-m RR: Non-inferiority and superiority tests

					Confidence interval		*p*-value	
Dialyzer	*N*	LS mean	SE	Level	Lower	Upper		
PP Population								
FX CorAL 600	69	76.28	1.45	95.0%	73.41	79.15		
FX CorDiax 600	67	75.69	1.45	95.0%	72.82	78.56		
xevonta Hi 15	69	74.48	1.45	95.0%	71.61	77.35		
Difference FX CorAL 600 vs. FX CorDiax 600		0.59	0.39	95.0%	-0.18	1.36	**< 0.0001** ^**1**^	0.0658^2^
				97.5%	-0.29	1.47		
Difference FX CorAL 600 vs. xevonta Hi 15		1.79	0.38	95.0%	1.03	2.55	**< 0.0001** ^**1**^	**< 0.0001** ^**2**^
				97.5%	0.92	2.66		
ITT Population								
FX CorAL 600	72	76.31	1.57	95.0%	73.20	79.42		
FX CorDiax 600	67	75.71	1.58	95.0%	72.59	78.82		
xevonta Hi 15	72	74.49	1.57	95.0%	71.38	77.60		
Difference FX CorAL 600 vs. FX CorDiax 600		0.60	0.39	95.0%	-0.16	1.37	**< 0.0001** ^**1**^	0.0606^2^
				97.5%	-0.27	1.48		
Difference FX CorAL 600 vs. xevonta Hi 15		1.82	0.38	95.0%	1.06	2.57	**< 0.0001** ^**1**^	**< 0.0001** ^**2**^
				97.5%	0.95	2.69		

^1^ p-value to conclude non-inferiority; ^2^ p-value to conclude superiority; LS mean: least squares mean; N: number of patients; SE: standard error

### Outcomes – secondary endpoints: performance

Table 6 shows the secondary performance endpoints. β2-m clearance of FX CorAL was superior vs. xevonta (*p* < 0.001) and higher vs. FX CorDiax, albeit not statistically significant (*p* = 0.539). Regarding RR and clearance of myoglobin, the second middle molecule, FX CorAL was superior to both comparators (*p* < 0.001 for all analyses). RRs and clearances of the small molecules phosphate, creatinine, and urea were comparable between dialyzers.

**Table 6:** Secondary performance endpoints: Descriptive statistics and superiority tests

	LS mean			LS mean difference [95% CI]			*p*-value			
Variable	FX CorAL 600	FX CorDiax 600	xevonta Hi 15	FX CorAL 600 vs. FX CorDiax 600	FX CorAL 600 vs. xevonta Hi 15	FX CorDiax 600 vs. xevonta Hi 15	Overall	FX CorAL 600 vs. FX CorDiax 600	FX CorAL 600 vs. xevonta Hi 15	FX CorDiax 600 vs. xevonta Hi 15
β2-m clearance [mL/min]	111.09	110.13	102.54	0.96 [-2.13, 4.05]	8.55 [5.51, 11.59]	7.59 [4.51, 10.67]	**< 0.001**	0.539	**< 0.001**	**< 0.001**
Myoglobin removal rate [%]	62.37	58.09	51.21	4.28 [2.34, 6.21]	11.16 [9.25, 13.07]	6.88 [4.94, 8.82]	**< 0.001**	**< 0.001**	**< 0.001**	**< 0.001**
Myoglobin clearance [mL/min]	69.98	63.50	49.07	6.48 [3.36, 9.60]	20.91 [17.84, 23.98]	14.43 [11.32, 17.54]	**< 0.001**	**< 0.001**	**< 0.001**	**< 0.001**
Phosphate removal rate [%]	61.03	60.82	61.55	0.21 [-1.70, 2.11]	-0.53 [-2.41, 1.35]	-0.73 [-2.64, 1.17]	0.733	0.831	0.579	0.448
Phosphate clearance [mL/min]	189.23	192.84	194.87	-3.60 [-10.74, 3.53]	-5.63 [-12.66, 1.39]	-2.03 [-9.14, 5.08]	0.278	0.319	0.115	0.573
Creatinine removal rate [%]	68.80	68.77	69.60	0.03 [-1.08, 1.15]	-0.79 [-1.90, 0.31]	-0.83 [-1.95, 0.29]	0.252	0.954	0.157	0.147
Creatinine clearance [mL/min]	182.34	186.50	187.65	-4.16 [-11.11, 2.78]	-5.31 [-12.15, 1.53]	-1.15 [-8.07, 5.78]	0.276	0.238	0.127	0.744
Urea removal rate [%]	76.68	76.54	77.64	0.14 [-0.94, 1.21]	-0.96 [-2.02, 0.10]	-1.09 [-2.17, -0.02]	0.091	0.801	0.076	**0.046**
Urea clearance [mL/min]	192.67	196.18	198.25	-3.50 [-10.91, 3.90]	-5.58 [-12.89, 1.74]	-2.07 [-9.48, 5.34]	0.317	0.351	0.134	0.581

^1^ p-value to conclude difference in means. AUC: area under the curve; BL: baseline; CI: confidence interval; LS mean: least squares mean; N: number of patients. p-values < 0.05 given in bold. p-values are not adapted for multiple testing

### Outcomes – secondary endpoints: Hemocompatibility

**Fig. 2:**
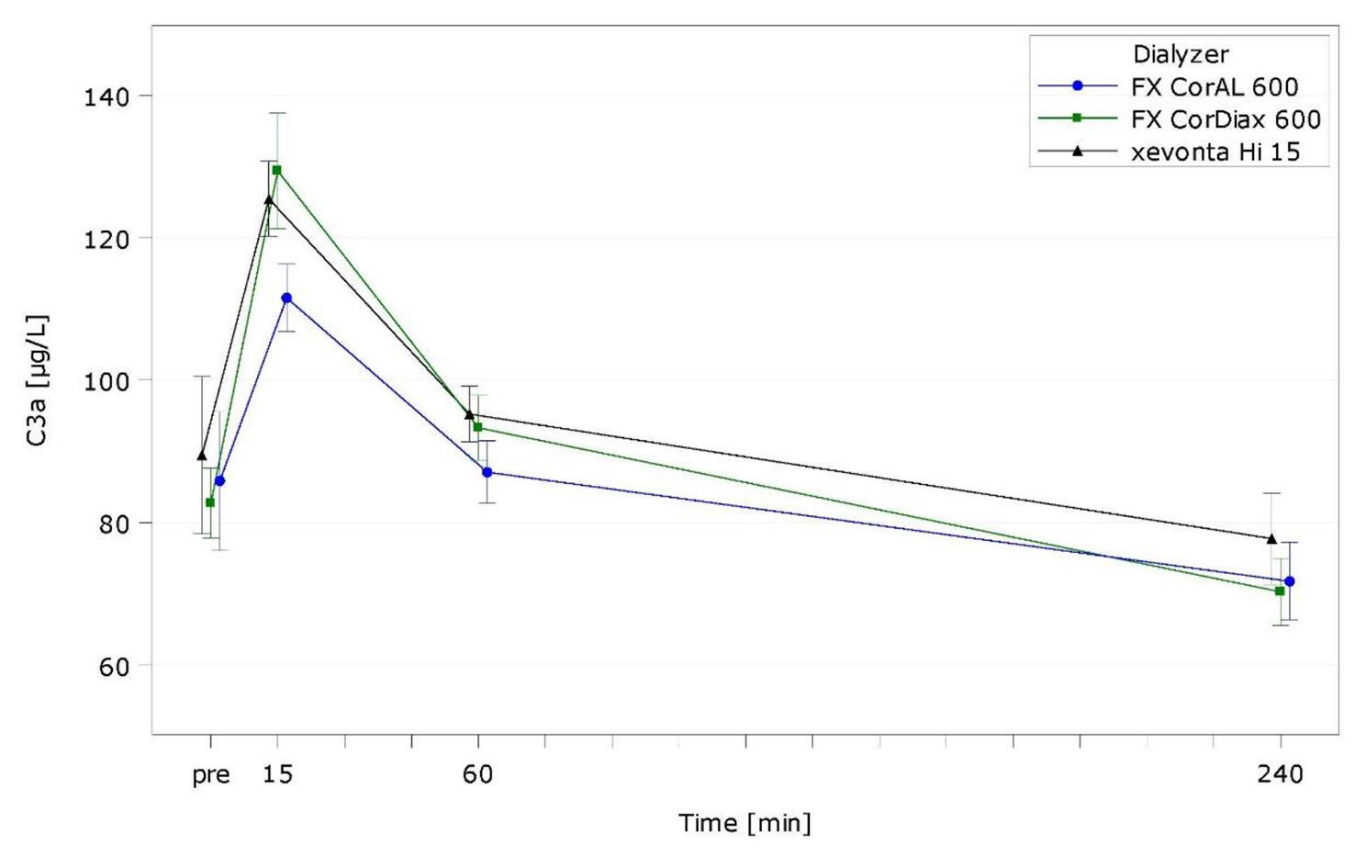
C3a over time by dialyzer (ITT population, LS mean)

**Fig. 3:**
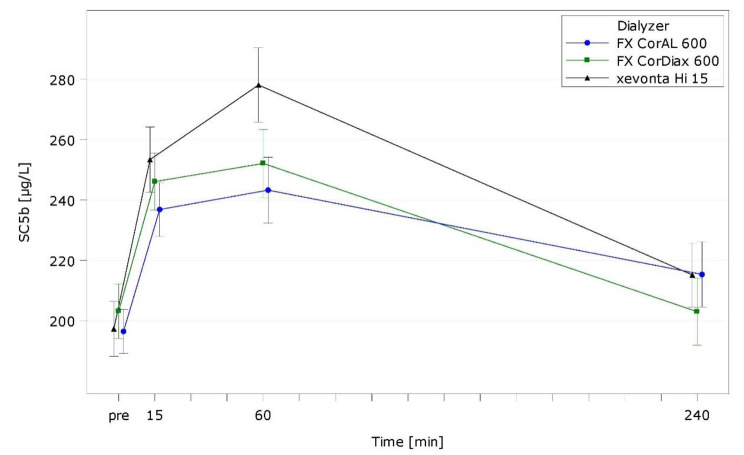
sC5b-9 over time by dialyzer (ITT population, LS mean)

**Fig. 4:**
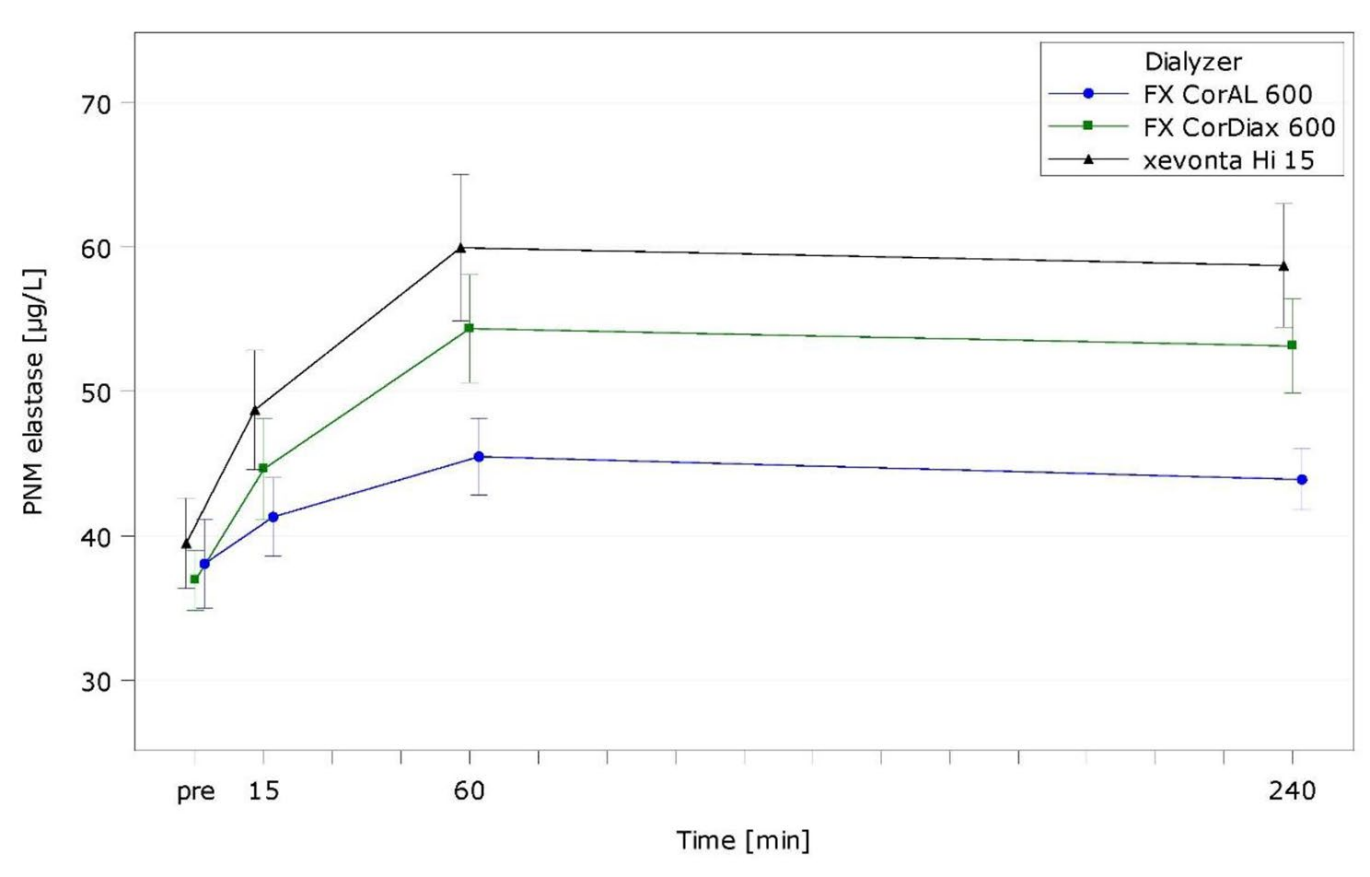
Polymorphonuclear (PMN) Elastase over time by dialyzer (ITT population, LS mean)

**Fig. 5:**
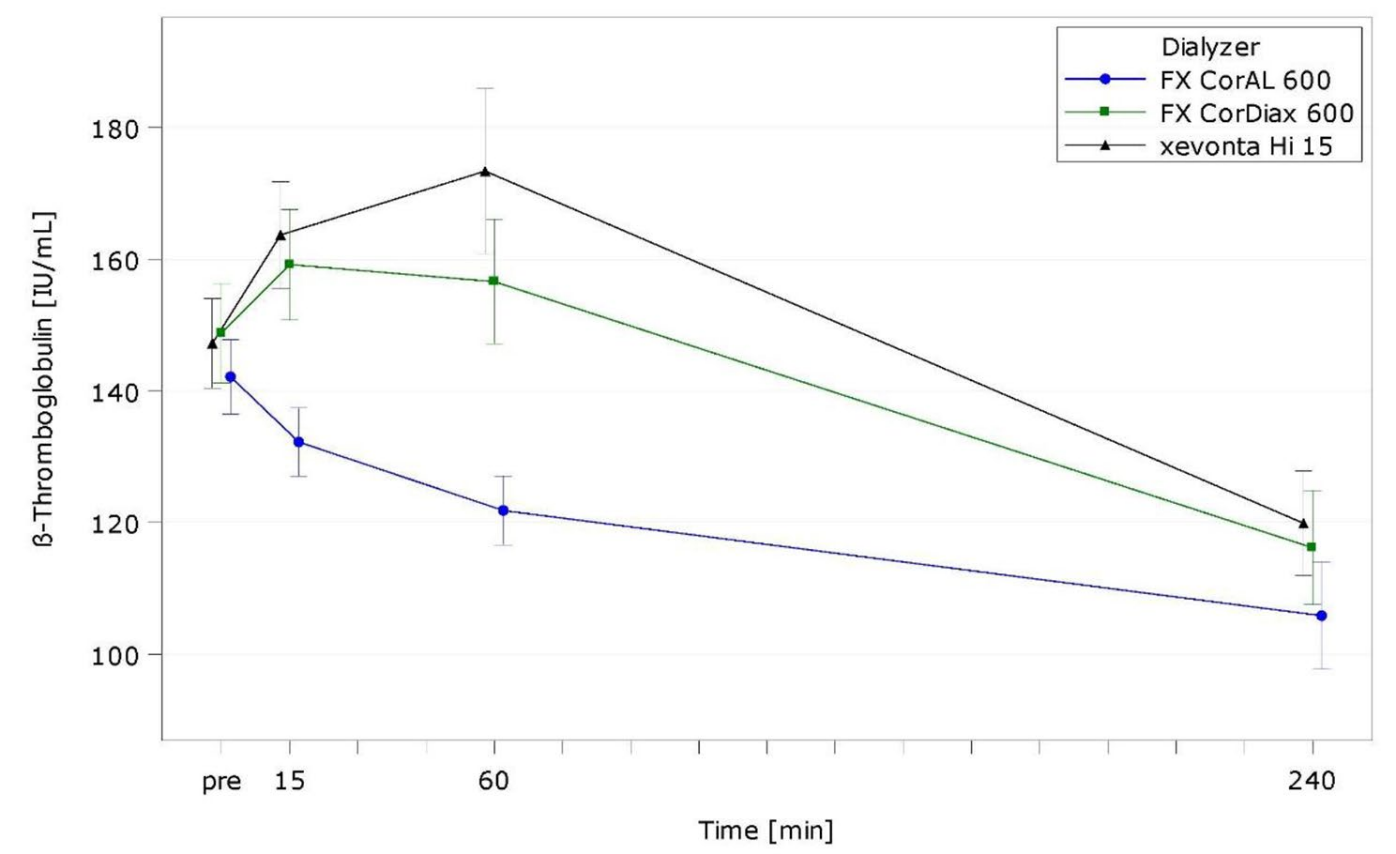
β-Thromboglobulin (β-TG) over time by dialyzer (ITT population, LS mean)

Table S4 presents intra- and interdialytic results of hemocompatibility markers. Figures 2, 3 and 4, and Fig. 5 show representative markers for complement activation (C3a; sC5b-9), cell activation/inflammation (PMN elastase), and platelet activation (β-TG).

### Intradialytic changes

#### Complement activation

With all dialyzers, complement factors peaked rapidly after 15 min (C3a) or 60 min (sC5b-9) and decreased until end of treatment thereafter (Figs. 2 and 3). For both complement factors, the increase at peak levels was lowest for FX CorAL (C3a: *p* = 0.0034 vs. FX CorDiax, *p* = 0.0287 vs. xevonta; sC5b-9: *p* = 0.3371 vs. FX CorDiax, *p* < 0.0001 vs. xevonta).

#### Cell activation/inflammation

Leukocytes showed a typical drop 15 min after treatment start with all dialyzers. FX CorAL presented the lowest drop (*p* = 0.0150 vs. FX CorDiax, *p* = 0.2456 vs. xevonta), which was mainly linked to the lowest drop in monocytes (*p* = 0.5326 vs. FX CorDiax, *p* = 0.0116 vs. xevonta) and neutrophils (*p* = 0.0191 vs. FX CorDiax, *p* = 0.2576 vs. xevonta). Cell activation markers LTB-4 and PMN elastase also increased until 15 min (LTB-4) or 60 min (PMN elastase; Fig. 4) with all dialyzers and showed lowest rise with FX CorAL (LTB-4: *p* = 0.0470 vs. FX CorDiax, *p* = 0.5391 vs. xevonta; PMN elastase: *p* = 0.0031 vs. FX CorDiax, *p* = 0.0003 vs. xevonta). The cytokine concentrations (IL-6, IL-8) were near the limit of detection with all dialyzers.

#### Platelet activation

With all dialyzers, platelets showed a typical drop 15 min after treatment start, least with FX CorAL (*p* = 0.2241 vs. FX CorDiax, *p* = 0.1329 vs. xevonta). Platelet activation marker β-TG displayed an initial increase with FX CorDiax and xevonta, which peaked after 15 min (FX CorDiax) or 60 min (xevonta) and declined afterwards (Fig. 5). FX CorAL evoked no initial β-TG peak (*p* < 0.0001 vs. both comparators at 60 min), but a constant decrease during treatment. TxB2 peaked at 15 min, with FX CorAL presenting the highest increase (*p* = 0.0959 vs. FX CorDiax, *p* = 0.0259 vs. xevonta).

#### Oxidative stress

Oxidative stress marker MDA showed a comparable continuous decrease with all dialyzers, whereas activity of the oxidative defense marker GSH-Px increased after treatment start and declined until termination for all dialyzers. However, standard deviations were high and results at the assays’ limits of detection.

### Interdialytic changes

Hemocompatibility markers showed neither conspicuous nor statistically significant changes over one HDF period, i.e., twelve HDF sessions with the same dialyzer type. Together with the lack of period, sequence, or carry over effects (see above), this indicates that the choice of wash-in phases and sampling sessions (see Figure S1) was adequate.

### Outcomes – secondary endpoints: PROs

PRO results per dialyzer are tabulated in Table S1. PSQI, KDQOL fatigue domain, peak PNRS and IRLSSG scores were comparable between dialyzers. Average PSQI scores indicate borderline poor sleep quality among participants. Average peak PNRS, fatigue, and IRLSSG scores indicate mild-to-no itching, reasonable vitality, and mild-to-no RLS among participants.

### Outcomes – secondary endpoints: adverse events

During the study, 64 patients (78.0%) experienced 219 adverse events (AEs). There were no obvious differences in overall AE numbers or profiles between dialyzers. Nine AEs in eight patients were possibly or probably related to a dialyzer. No SAE was considered related to a dialyzer. Additional clinical safety information is provided in **Table S5**.

## Discussion

The present clinical trial investigated the impact of increased hydrophilic membrane modification on performance and hemocompatibility among 82 patients on online post-dilution HDF. As investigational device, the trial included the novel FX CorAL dialyzer, which contains a hydrophilic membrane with increased PVP content on the blood-side surface. This study supports recent experimental studies which showed a strong correlation of hydrophilic membrane modification with reduced protein fouling, stabilized performance over time and the favorable hemocompatibility profile [9, 10, 17, 21].

In the present study, the FX CorAL showed the highest β2-m RR among the three investigated dialyzers (76.31%), followed by FX CorDiax (75.71%) and xevonta (74.49%). FX CorAL was significantly non-inferior to both comparators and significantly superior to xevonta. While the differences appear small, though statistically significant, the chronic and repeated nature of the dialysis treatment may multiply this effect over time. However, we cannot conclude from the present study whether these observed differences may translate into clinically significant long-term effects. Results for other markers confirmed the strong performance of FX CorAL, especially for middle molecules. FX CorAL showed highest RR of myoglobin compared to FX CorDiax and xevonta, with significant differences observed (*p* < 0.001 for all analyses). Notably, these differences appear even more pronounced for this larger molecule (17 kDa), with RR of 62.37%, (FX CorAL) vs. 58.09% (FX CorDiax) and 51.21% (xevonta), than for the smaller β2-m (12 kDa). Therefore, while not specifically analyzed in this study, FX CorAL’s performance benefits may increase with the size of middle molecules. Importantly, for albumin (66 kDa), FX CorAL shows low removal rates, supporting its safe use for HDF treatments [11]. Thus, future studies should consider including a broader range of middle molecule markers to provide further insights into FX CorAL’s performance benefits.

For all analyses, the ITT and the PP analyses showed corresponding results, confirming the internal validity of the data. In addition, the comPERFORM trial provides external validity to eMPORA III: RR and clearance of uremic toxins were highly similar for FX CorAL in both studies – under comparable conditions regarding treatment modality as well as flow and substitution rates [11].

The kinetics of complement activation (C3a, sC5b-9) confirm previous results from clinical studies and in vitro experiments, where FX CorAL showed the lowest complement activation among other dialyzers [19, 9, 34, 35, 20]. Lower complement activation as shown for FX CorAL is associated with a decrease in leukocyte activation [34, 35]. Accordingly, the initial drop of leukocyte counts, especially of monocytes and neutrophils, was lowest with FX CorAL as well as the release of PMN elastase and LTB-4 (eicosanoids), indicating lowest cell activation. Inflammatory markers IL-6 and IL-8 were at the limit of detection, probably due to low activation, slow synthesis, and efficient dialytic elimination [36, 37]. Additional markers implicated in vascular inflammation like formation of neutrophil extracellular traps (NETs) could have provided more insight into this process [35, 38, 39].

Different from in vitro recirculation studies with human blood, where FX CorAL showed the lowest drop in platelet counts as compared to all investigated dialyzers [10], the drop in platelets in eMPORA III was statistically not different between the three dialyzers. However, there was a clear differentiation in favor of FX CorAL regarding the release of β-thromboglobulin (β-TG). In a clinical setting, β-TG appears to be a more sensitive marker of platelet activation than the pure drop in platelet counts. Our β-TG results are in line with the PMN elastase kinetics; this supports the role of PMN in the formation of pro-thrombotic platelet-PMN complexes, reducing functional and circulating platelets [34, 35, 40]. Of note, while β-TG continuously decreased with FX CorAL, indicating low thrombotic activation, it showed an increase in the first hour of treatment with both comparators. In contrast, TxB2 as a second marker of platelet activation showed inconsistent findings, which could be caused by the strong elimination due to its low molecular weight (371 Da).

Markers of oxidative stress (MDA) and oxidative defense (GSH-Px) revealed similar results for all dialyzers, at the assays’ limits of detection and with high standard deviations. Thus, methodological limits do not permit an interpretation of differences between dialyzers.

Patient reported outcomes (PROs) showed no statistically significant differences between dialyzers. Nonetheless, the subjective perception that PRO symptoms such as sleep quality, pruritus, fatigue, and restless legs syndrome improve, may – despite short recall periods – require longer interventional exposure times, especially as physiological mechanisms influencing PROs are complex and mostly unknown [41–43]. Given eMPORA III recruited a stable and comparatively healthy patient population with minimal PRO symptoms, future PRO studies should [1] recruit populations exhibiting meaningful symptoms and [2] include longer treatment periods to overcome long-engrained behaviors like sleeping times, exercise habits, or diet.

Regarding safety, eMPORA III found no new signals and no conspicuous differences in overall AE numbers or profiles between dialyzers. AEs in all treatment periods – like dialytic hypotension or hypovolemia – were typical for a population receiving hemodialysis, as was one SAE of hyperkalemia and one case of dialyzer clotting.

eMPORA III narrowed the knowledge gap between controlled short-term investigations and non-controlled real-world observations collecting a less detailed set of variables [19, 20, 44]. The performance of FX CorAL was comparable to earlier investigations, and it was non-inferior to both comparators and superior to xevonta in the present study.

In line with *in-vitro* data, intradialytic hemocompatibility profiles in eMPORA III found differences between dialyzers, with FX CorAL’s modified ‘hydrolayer’-forming membrane generally showing lower complement, cell, and platelet activation [9, 10, 21]. Such positive effects of hydrophilic membrane modifications are described also by other reports, such as by Oshihara et al. [45], who investigated the NV membrane and demonstrated improved hemocompatibility in terms of leukocyte and platelet activation.

The present study provides further evidence that increased membrane hydrophilicity improves performance and hemocompatibility of dialyzers. Such membrane modifications, as included in the novel FX CorAL dialyzer, may also help to improve clinical outcomes in hemodialysis patients. Inflammation, atherosclerosis, and thrombosis lead to the well-described clinical endpoints of cardio- and cerebrovascular disease, the major cause behind the early death of patients on hemodialysis [4, 35, 46]. Thus, larger long-term investigations should examine whether FX CorAL may, in addition to surrogates, also improve cardiovascular and mortality endpoints.

### Electronic supplementary material

Below is the link to the electronic supplementary material.

Supplementary Material 1: Figure S1: Simplified study schedule: Change of dialyzers and blood sampling. Table S1: Methods for obtaining blood samples and analytical methods of investigated variables. Table S2: Overview of Patient Reported Outcomes (PRO; Safety population). Table S3: Anticoagulation by dialyzer (Safety population). Table S4: Overview of hemocompatibility markers: intra- and interdialytic changes (ITT population). Table S5 Overview of Serious Adverse Events (SAEs), Adverse Events (AEs), and clinical safety (Safety population). Sect. 1 Formulas for calculating β2-m Removal Rate (RR) and bloodside clearances K_b_; Statistical concept

## Data Availability

Aggregated data underlying this article are available in the article and in its online supplementary material. Personal data underlying this article cannot be shared publicly to maintain the privacy of individuals that participated in the study.
