# Supplementary material for: Randomized investigation of increased dialyzer membrane hydrophilicity on hemocompatibility and performance
Source: BMC Nephrol. 2024 Jul 10;25:220. doi: 10.1186/s12882-024-03644-5 (PMC11234537; doi:10.1186/s12882-024-03644-5)
Supplement: Supplementary file 1 — Supplementary Material 1: Figure S1: Simplified study schedule: Change of dialyzers and blood sampling. Table S1: Methods for obtaining blood samples and analytical methods of investigated variables. Table S2: Overview of Patient Reported Outcomes (PRO; Safety population). Table S3: Anticoagulation by dialyzer (Safety population). Table S4: Overview of hemocompatibility markers: intra- and interdialytic changes (ITT population). Table S5 Overview of Serious Adverse Events (SAEs), Adverse Events (AEs), and clinical safety (Safety population). Sect. 1 Formulas for calculating β2-m Removal Rate (RR) and bloodside clearances Kb; Statistical concept [file 12882_2024_3644_MOESM1_ESM.pdf]

eMPORA III: Randomized comparison of dialyzer performance and hemocompatibility during hemodiafiltration

Götz Ehlerding, Wolfgang Ries, Manuela Kempkes-Koch, Ekkehard Ziegler, Petra Ronová, Mária Krizsán, Jana Verešová, Mária Böke, Ansgar Erlenkötter, Robert Nitschel, Adam M. Zawada, James P. Kennedy, Jennifer Braun, John W. Larkin, Natalia Korolev, Thomas Lang, Bertram Ottillinger, Manuela Stauss-Grabo and Bettina Griesshaber*

*Corresponding author:
Dr. Bettina Griesshaber
Fresenius Medical Care Deutschland GmbH
Global Biomedical Evidence Generation
Global Medical Office
Else-Kröner-Straße 1
61352 Bad Homburg, Germany
P +49 6172 268 6766
Bettina.Griesshaber@freseniusmedicalcare.com

Supplement – Table of Contents

Supplemental Figure S1: Simplified study schedule: Change of dialyzers and blood sampling

Supplemental Table S1: Methods for obtaining blood samples and analytical methods of investigated variables

Supplemental Table S2: Overview of Patient Reported Outcomes (PRO; Safety population)

Supplemental Table S3: Anticoagulation by dialyzer (Safety population)

Supplemental Table S4: Overview of hemocompatibility markers: intra- and interdialytic changes (ITT population)

Supplemental Table S5: Overview of Serious Adverse Events (SAEs), Adverse Events (AEs), and clinical safety (Safety population)

Supplemental Section 1: Formulas for calculating β2-m Removal Rate (RR) and bloodside clearances K_b; Statistical concept

Figure S1: Simplified study schedule: Change of dialyzers and blood sampling

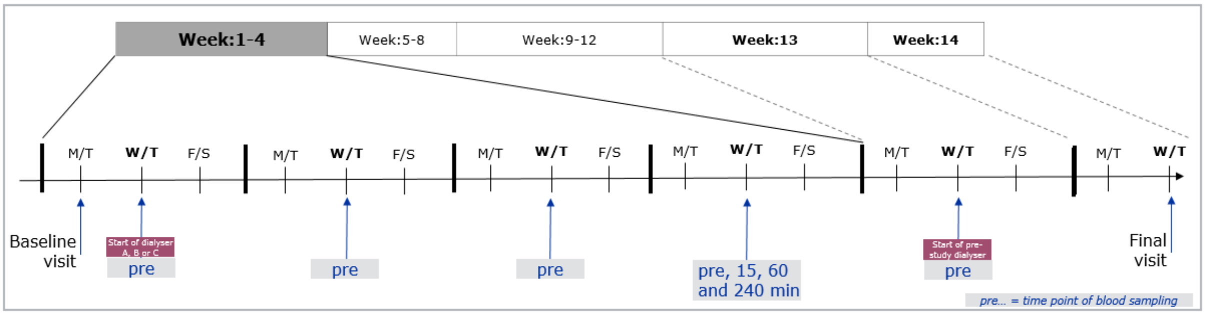

M/T: Monday or Tuesday; W/T: Wednesday or Thursday; F/S: Friday or Saturday

Table S1: Methods for obtaining blood samples and analytical methods of investigated variables

**Methods for obtaining blood samples**<br>• The pre-dialysis blood sample was obtained from the native arterial needle, before it was flushed or connected to the blood tubing. The venous blood samples were drawn slowly from the venous port – before the blood was diluted with the substitution solution – at the standard blood flow of approx. 300 mL/min, at running blood and dialysate. Arterial blood samples at the later timepoints were drawn at a temporarily reduced blood flow of 100 mL/min and at running dialysate.
**Analytical methods of investigated variables**<br>• Creatinine: COBAS INTEGRA 400 plus analyzer (Roche, Mannheim, Germany) using CREJ2 method (Jaffé)<br>• Urea: COBAS INTEGRA 400 plus analyzer (Roche, Mannheim, Germany) using UREAL method<br>• Phosphate: COBAS INTEGRA 400 plus analyzer (Roche, Mannheim, Germany) using PHOS2 method<br>• β2-microglobulin: COBAS INTEGRA 400 plus analyzer (Roche, Mannheim, Germany) using Tina-quant β2-microglobulin method<br>• Myoglobin: COBAS INTEGRA 400 plus analyzer (Roche, Mannheim, Germany) using MYO2 method<br>• C3a: C3a Plus MicroVue immunoassay (Quidel, San Diego, USA)<br>• sC5b-9: sC5b-9 Plus MicroVue immunoassay (Quidel, San Diego, USA)<br>• Hemoglobin, WBC/Leukocytes, PLT count: XN-1000 Pure analyzer (Sysmex, Kobe, Japan)<br>• PMN elastase: PMN Elastase Human ELISA Kit (Thermo Fisher Scientific, Waltham, USA)<br>• IL-6: Human IL-6 Quantikine ELISA Kit D6050 (R&D Systems, Minneapolis, USA)<br>• IL-8: Human IL-8/CXCL8 Quantikine ELISA Kit D8000C (R&D Systems, Minneapolis, USA)<br>• LTB-4: LTB4 Parameter Assay Kit KGE006B (R&D Systems, Minneapolis, USA)<br>• sICAM-1: Human ICAM-1/CD54 Non-Allele-specific Quantikine ELISA Kit DCIM00 (R&D Systems, Minneapolis, USA)<br>• hsCRP: COBAS INTEGRA 400 plus analyzer (Roche, Mannheim, Germany) using CRPHS method<br>• β-TG: Asserachrom β-TG test (Diagnostica Stago, Asnières-sur-Seine, France)<br>• TxB2: Thromboxane B2 Parameter Assay Kit KGE011 (R&D Systems, Minneapolis, USA)<br>• MDA: Ultimate 3000 HPLC FLD (Thermo Fisher Scientific, Waltham, USA)<br>• GSH-Px activity: Glutathione Peroxidase (GSH-Px) Activity Assay Kit E-bC-K096-M-96 (Elabscience, Houston, USA)

Table S2: Overview of Patient Reported Outcomes (PRO; Safety population)

		FX CorAL 600 (N=74)	FX CorDiax 600 (N=71)	xevonta Hi 15 (N=76)	Total (N=218)
**Pittsburgh Sleep Quality Index (PSQI) by dialyzer**					
**PSQI**	**n**	71	71	76	218
	**Mean±SD**	5.8±3.75	5.8±3.77	5.6±3.83	5.7±3.77
	**Min, Median, Max**	0.0, 5.0, 17.0	0.0, 6.0, 17.0	0.0, 5.0, 19.0	0.0, 5.0, 19.0
	**LS mean difference [95% CI]**	**FX CorAL vs.**	-0.07 [-0.54, 0.39]	-0.02 [-0.48, 0.44]	N/A
	**p-value**	**FX CorAL vs.**	0.7508	0.9346	N/A
**Kidney Disease Quality of Life (KDQOL) Instrument Short Form v1.3**					
**KDQOL v1.3 "Fatigue domain"**	**n**	71	71	76	218
	**Mean±SD**	63.7±20.35	64.2±22.78	63.6±21.32	63.8±21.41
	**Min, Median, Max**	10.0, 65.0, 100.0	0.0, 60.0, 100.0	10.0, 60.0, 100.0	0.0, 65.0, 100.0
	**LS mean difference [95% CI]**	**FX CorAL vs.**	-0.10 [-3.56, 3.36]	0.30 [-3.11, 3.72]	N/A
	**p-value**	**FX CorAL vs.**	0.9530	0.8605	N/A
**Pruritus Numerical Rating Scale (PNRS) by dialyzer**					
**Peak PNRS**	**n**	71	71	76	218
	**Mean±SD**	0.9±2.03	1.1±1.92	0.9±1.89	1.0±1.94
	**Min, Median, Max**	0.0, 0.0, 10.0	0.0, 0.0, 8.0	0.0, 0.0, 7.0	0.0, 0.0, 10.0
	**LS mean difference [95% CI]**	**FX CorAL vs.**	-0.08 [-0.42, 0.27]	0.08 [-0.26, 0.42]	N/A
	**p-value**	**FX CorAL vs.**	0.6558	0.6506	N/A
**International Restless Legs Syndrome Study Group (IRLSSG) Rating Scale by dialyzer**					
**IRLSSG**	**n**	71	71	76	218
	**Mean±SD**	3.4±6.66	3.2±5.89	2.9±6.24	3.2±6.24
	**Min, Median, Max**	0.0, 0.0, 28.0	0.0, 0.0, 19.0	0.0, 0.0, 25.0	0.0, 0.0, 28.0
	**LS mean difference [95% CI]**	**FX CorAL vs.**	0.24 [-0.83 1.32]	0.33 [-0.73 1.39]	N/A
	**p-value**	**FX CorAL vs.**	0.6533	0.5370	N/A

Legend: Max: maximum; Min: minimum; LS mean: Least Squares mean; N: number of patients; n: number of patients with data available; SD, standard deviation

LS mean difference shows the difference in the change in the PRO from baseline to the end of each period in reference to the FX CorAL 600 dialyzer (FX CorAL 600 vs. FX CorDiax 600, and FX CorAL 600 vs. xevonta Hi 15) with the and 95% confidence interval. The p-value indicates the level of statistical significance for the LS mean difference.

Questionnaires were collected at baseline at study start and at the end of each subsequent period, i.e., after patients had received 12 HDF sessions with each dialyzer. The questionnaires included:

Pittsburgh Sleep Quality Index (PSQI) [1]

- PRO assessment: perceived sleep quality
- Range: 0 (best sleep quality) to 21 (poorest sleep quality)
- Recall period: previous month

Kidney Disease Quality of Life (KDQOL) questionnaire v1.3, fatigue domain questions 9a, 9e, 9g, and 9i [2]

- PRO assessment: perceived vitality/energy level
- Range: 0 (lowest vitality/energy) to 100 (highest vitality/energy)
- Recall period: previous month

Peak Pruritus Numerical Rating Scale (PNRS) [3–6]

- PRO assessment: perceived severity of worst itch
- Range: 0 (no itch) to 10 (worst itch imaginable)
- Recall period: previous 24 hours

International Restless Legs Syndrome Study Group Rating Scale (IRLSSG) [7–9]

- PRO assessment: perceived severity/impact of restless legs
- Range total score: 0 (no restless legs) to 40 (very severe restless legs)
- Recall period: previous week

Table S3: Anticoagulation by dialyzer (Safety population)

		Dialyzer		
		FX CorAL 600 (N=74)	FX CorDiax 600 (N=71)	xevonta Hi 15 (N=76)
Anticoagulation				
Clexane Bolus	[n (%)]	6 (8.1%)	5 (7.0%)	5 (6.6%)
	[dose (IU)]	3833 ± 1835	4200 ± 1789	4200 ± 1789
Non-Clexane LMWH Bolus	[n (%)]	20 (27.0%)	19 (26.8%)	21 (27.6%)
	[dose (IU)]	5493 ± 2129	5482 ± 2186	5593 ± 2125
Non-Clexane LMWH – 2 doses	[n (%)]	1 (1.4%)	1 (1.4%)	1 (1.3%)
	Bolus [dose (IU)]	8000	8000	8000
	Infusion [dose (IU)]	1000	1000	1000
UF heparin	[n (%)]	47 (63.5%)	46 (64.8%)	48 (63.2%)
	Bolus [dose (IU)]	2723 ± 1664	2663 ± 1606	2729 ± 1644
	Infusion [dose (IU)]	1447 ± 910.2	1486 ± 913.0	1469 ± 901.5

IU: International Units; LMWH: Low Molecular Weight Heparin; UF: Unfractionated.
Doses are presented as mean ± SD

Table S4: Overview of hemocompatibility markers: intra- and interdialytic changes (ITT population)

Variable (molecular weight) [unit]	Key indicator	INTRA					INTER		
		FX Coral 600	FX CorDiax 600	xevonta Hi 15	FX Coral vs FX CorDiax	FX Coral vs xevonta	FX Coral 600	FX CorDiax 600	xevonta Hi 15
		LS mean			p value	p value	p value		
Complement Activation									
**C3a (9 kDa) [µg/L]**	15 - pre	23.99	42.18	37.33	**0.0034**	**0.0287**	0.8331	0.7589	0.6983
	60 - pre	-0.52	5.70	7.13	0.3158	0.2095			
	240 - pre	-15.86	-16.51	-10.37	0.9159	0.3648			
	AUC - pre	-8.21	15.68	10.83	0.6069	0.6741			
**sC5b-9 (1000 kDa) [µg/L]**	15 - pre	38.51	45.47	53.42	0.3133	**0.0283**	0.9440	0.9842	0.6660
	60 - pre	45.50	52.13	78.16	0.3371	**<0.0001**			
	240 - pre	17.06	0.48	15.21	**0.0162**	0.7845			
	AUC - pre	128.60	119.20	192.15	0.7161	**0.0126**			
Cell activation / Inflammation									
**WBC (leukocytes) [10^3/µL]**	15 - pre	-0.56	-0.81	-0.67	**0.0150**	0.2456	0.9646	0.5769	0.7128
	60 - pre	-0.30	-0.21	-0.29	0.3837	0.9464			
	240 - pre	-1.00	-0.84	-0.76	0.1141	**0.0139**			
	AUC - pre	-2.17	-1.88	-2.10	0.3880	0.8282			
**Monocytes [10^3/µL]**	15 - pre	-0.20	-0.22	-0.25	0.5326	**0.0116**	0.6594	0.8033	0.6311
	60 - pre	-0.16	-0.17	-0.17	0.4999	0.6630			
	240 - pre	-0.26	-0.23	-0.20	0.1084	**0.0017**			
	AUC - pre	-0.77	-0.74	-0.71	0.5658	0.2817			
**Lymphocytes [10^3/µL]**	15 - pre	-0.15	-0.20	-0.16	0.0887	0.8665	0.6443	0.7618	0.8363
	60 - pre	-0.19	-0.23	-0.22	0.1618	0.3663			
	240 - pre	-0.15	-0.15	-0.12	0.9418	0.2596			
	AUC - pre	-0.69	-0.72	-0.64	0.7619	0.6770			
**Neutrophiles [10^3/µL]**	15 - pre	-0.15	-0.36	-0.25	**0.0191**	0.2576	0.9418	0.5225	0.6240
	60 - pre	0.10	0.23	0.12	0.1529	0.8862			
	240 - pre	-0.50	-0.39	-0.39	0.1894	0.2001			
	AUC - pre	-0.52	-0.21	-0.57	0.2557	0.8489			
**Eosinophiles [10^3/µL]**	15 - pre	-0.03	-0.04	-0.03	0.1950	0.3180	0.8403	0.5775	0.5077
	60 - pre	-0.02	-0.03	-0.03	0.1487	0.1970			
	240 - pre	-0.07	-0.07	-0.06	0.6110	0.0932			
	AUC - pre	-0.15	-0.18	-0.15	0.1686	0.6787			
**Basophiles [10^3/µL]**	15 - pre	-0.01	0.00	-0.01	0.0846	0.9949	0.8645	0.8976	0.8951
	60 - pre	-0.01	0.00	-0.01	0.3758	0.7125			
	240 - pre	-0.01	0.01	-0.01	0.3312	0.5988			
	AUC - pre	-0.03	-0.02	-0.03	0.3128	0.8955			
**PMN elastase (30 kDa) [µg/L]**	15 - pre	6.42	11.57	10.19	0.1193	0.2512	0.2030	0.3892	0.0987
	60 - pre	11.21	20.92	22.93	**0.0031**	**0.0003**			
	240 - pre	9.81	19.53	21.74	**0.0030**	**0.0002**			
	AUC - pre	40.9	61.30	71.05	0.1634	**0.0361**			

Variable (molecular weight) [unit]	Key indicator	INTRA					INTER		
		FX Coral 600	FX CorDiax 600	xevonta Hi 15	FX Coral vs FX CorDiax	FX Coral vs xevonta	FX Coral 600	FX CorDiax 600	xevonta Hi 15
		LS mean			p value	p value	p value		
**IL-6 (22-28 kDa) [pg/mL]**	15 - pre	-0.83	-0.60	-0.46	0.5947	0.3864	0.8061	0.4939	0.3074
	60 - pre	-1.28	-0.90	-1.02	0.3718	0.5301			
	240 - pre	-1.31	-0.66	-0.68	0.1288	0.1308			
	AUC - pre	-4.21	-2.96	-3.43	0.2945	0.5002			
**IL-8 (8-9 kDa) [pg/mL]**	15 - pre	-1.52	-1.94	-1.36	0.3756	0.7388	0.5802	0.4389	0.8236
	60 - pre	-3.15	-3.74	-3.24	0.2117	0.8392			
	240 - pre	-3.44	-4.48	-4.27	**0.0285**	0.0743			
	AUC - pre	-11.45	-13.36	-13.10	0.3710	0.4167			
**LTB-4 (336 Da) [pg/mL]**	15 - pre	160.6	219.4	178.4	**0.0470**	0.5391	N/A	N/A	N/A
	60 - pre	61.3	70.5	59.2	0.7553	0.9428			
	240 - pre	9.7	4.8	4.8	0.8686	0.8652			
	AUC - pre	200.4	228.0	190.1	0.5880	0.8364			
**sICAM-1 (58 kDa) [µg/L]**	15 - pre	11.56	6.61	3.68	0.4208	0.1929	0.3593	0.9772	0.6836
	60 - pre	0.05	0.39	7.93	0.9564	0.1934			
	240 - pre	-10.05	-3.34	-4.40	0.2736	0.3480			
	AUC - pre	-7.54	19.60	14.69	0.2285	0.3111			
**hsCRP (25 kDa) [mg/L]**	N/A	N/A	N/A	N/A	N/A	N/A	0.6172	0.5612	0.8872
Platelet Count / Activation									
**PLT [10^3/µL]**	15 - pre	-20.3	-23.9	-24.7	0.2241	0.1329	0.7218	0.5173	0.8168
	60 - pre	-25.2	-26.6	-29.4	0.6375	0.1505			
	240 - pre	-34.0	-33.8	-31.9	0.9406	0.4719			
	AUC - pre	-109.1	-108.8	-108.6	0.9756	0.9563			
**β-TG (36 kDa) [IU/mL]**	15 - pre	-12.5	10.9	16.6	**0.0034**	**0.0002**	0.6548	0.3104	0.2903
	60 - pre	-23.6	7.6	26.3	**<0.0001**	**<0.0001**			
	240 - pre	-38.5	-31.8	-27.2	0.4020	0.1496			
	AUC - pre	-96.4	-32.6	12.8	**0.0286**	**0.0002**			
**TxB2 (371 Da) [µg/L]**	15 - pre	0.66	0.42	0.34	0.0959	**0.0259**	0.4976	0.2590	0.9879
	60 - pre	-0.26	-0.23	-0.41	0.8589	0.2762			
	240 - pre	-1.04	-1.09	-1.09	0.7395	0.7517			
	AUC - pre	-1.80	-1.58	-2.07	0.7026	0.6365			
Oxidative Stress									
**MDA (72 Da)**	15 - pre	-0.02	-0.02	-0.03	0.9709	0.8214	0.8387	0.8336	0.7801
	60 - pre	-0.07	-0.06	-0.06	0.7674	0.7201			
	240 - pre	-0.08	-0.10	-0.08	0.4207	0.9878			
	AUC - pre	-0.22	-0.28	-0.24	0.3310	0.7488			
**GSH-Px activity (22 kDa)**	15 - pre	8.1	33.2	46.1	0.1203	**0.0174**	0.9073	0.6039	0.1869
	60 - pre	21.2	6.5	29.7	0.3646	0.5911			
	240 - pre	-8.1	-0.2	-22.9	0.6255	0.3486			
	AUC - pre	1.7	35.6	75.6	0.5816	0.2192			

Values indicate LS mean differences to the baseline (pre) values before the start of HDF. LS mean: Least Squares mean. Intradialytic: changes within a dialysis session. Interdialytic: changes over a dialysis period (12 sessions under one allocated dialyzer type). Shaded lines indicate parameters of key clinical interest for the variable. p-values formatted in bold script mark values <0.05 and the associated parameter. p-values are descriptive and not adjusted for multiple testing.

Table S5: Overview of Serious Adverse Events (SAEs), Adverse Events (AEs), and clinical safety (Safety population)

MedDRA SOC/PT	FX CorAL 600	FX CorDiax 600	xevonta Hi 15
**Any SAE**	2 (2.4) 2	4 (4.9) 5	3 (3.7) 3
Injury, poisoning and procedural complications	0 (0.0) 0	1 (1.2) 1	1 (1.2) 1
Femoral neck fracture	0 (0.0) 0	1 (1.2) 1	0 (0.0) 0
Lower limb fracture	0 (0.0) 0	0 (0.0) 0	1 (1.2) 1
Cardiac disorders	0 (0.0) 0	0 (0.0) 0	1 (1.2) 1
Acute myocardial infarction	0 (0.0) 0	0 (0.0) 0	1 (1.2) 1
General disorders and administration site conditions	0 (0.0) 0	1 (1.2) 1	0 (0.0) 0
Sudden cardiac death	0 (0.0) 0	1 (1.2) 1	0 (0.0) 0
Infections and infestations	0 (0.0) 0	1 (1.2) 2	0 (0.0) 0
Cardiac valve abscess	0 (0.0) 0	1 (1.2) 1	0 (0.0) 0
Endocarditis	0 (0.0) 0	1 (1.2) 1	0 (0.0) 0
Metabolism and nutrition disorders	0 (0.0) 0	0 (0.0) 0	1 (1.2) 1*
Hyperkalemia	0 (0.0) 0	0 (0.0) 0	1 (1.2) 1*
Renal and urinary disorders	1 (1.2) 1	0 (0.0) 0	0 (0.0) 0
Cystitis hemorrhagic	1 (1.2) 1	0 (0.0) 0	0 (0.0) 0
Respiratory, thoracic and mediastinal disorders	0 (0.0) 0	1 (1.2) 1	0 (0.0) 0
Dyspnea	0 (0.0) 0	1 (1.2) 1	0 (0.0) 0
Surgical and medical procedures	1 (1.2) 1	0 (0.0) 0	0 (0.0) 0
Renal transplant	1 (1.2) 1	0 (0.0) 0	0 (0.0) 0
**Any non-serious AE**	4 (4.9) 4	3 (3.7) 4	1 (1.2) 1
Vascular disorders	2 (2.4) 2	1 (1.2) 1	1 (1.2) 1
Dialysis hypotension	2 (2.4) 2	1 (1.2) 1	1 (1.2) 1
Product issues	1 (1.2) 1	1 (1.2) 1	0 (0.0) 0
Thrombosis in device	1 (1.2) 1	1 (1.2) 1	0 (0.0) 0
Gastrointestinal disorders	0 (0.0) 0	1 (1.2) 1	0 (0.0) 0
Vomiting	0 (0.0) 0	1 (1.2) 1	0 (0.0) 0
Metabolism and nutrition disorders	0 (0.0) 0	1 (1.2) 1	0 (0.0) 0
Hypovolaemia	0 (0.0) 0	1 (1.2) 1	0 (0.0) 0
Skin and subcutaneous tissue disorders	1 (1.2) 1	0 (0.0) 0	0 (0.0) 0
Pruritus	1 (1.2) 1	0 (0.0) 0	0 (0.0) 0
Overall N=82	Numbers indicate: No. of subjects (% of subjects) No. of events		
**Clinical safety**			
Repeated measurements of routine lab data and vital signs showed the patterns expected in patients undergoing state-of-the-art HDF. The mean hemoglobin concentration increased during HDF sessions (+0.6 g/dL overall), blood pressure decreased (systolic: -6.1 mmHg; diastolic: -2.2 mmHg), heart rate remained stable (+0.1 bpm), and body weight decreased (-2.1 kg). There were no conspicuous differences between dialyzers.			

HDF: Hemodiafiltration; MedDRA: Medical Dictionary for Regulatory Activities; PT: Preferred Term; SOC: System Organ Class.
* Related to the medical procedure. All other SAEs were neither considered related to the dialyzer nor to the medical procedure.

Section 1: Formulas for calculating β2-m Removal Rate (RR) and bloodside clearance K_b; Statistical concept

β2-m RR [%] was calculated using the following formula[10]:

$$\beta 2\text{-m RR} = \left[1 - \frac{(1-Hct_{240\min})}{(1-Hct_{pre})}\frac{C_{ß2m,240\min}}{C_{ß2m,pre}}\frac{Hct_{pre}}{Hct_{240\min}}\right] * 100$$

$Hct_{240\,min}$ denotes the hematocrit as a proportion after 240 min of HDF, Hct_{pre} the hematocrit as a proportion at the start of HDF; $c_{β2m,240\,min}$ denotes the β2-m concentration after 240 min of HDF, $c_{β2m,pre}$ the β2-m concentration at the start of HDF. Thus, β2-m RR was corrected for concentration effects due to the dialysis treatment by considering the hematocrit.

Removal rates of other molecules (secondary performance endpoints) were calculated according to the same formula, using the concentrations of the molecule at the start and after 240 min of HDF.

Bloodside clearances K_b [mL/min] were calculated using the following formula (adapted from[11]):

$$K_b = \frac{C_{art} \times Q_b(1-Hct) - C_{ven}(Q_b(1-Hct) - (Q_{sub} + Q_{UF}))}{C_{art}}$$

Hct denotes the arterial hematocrit as a proportion, Q_b [mL/min] the effective blood flow rate at the time clearance is calculated, Q_{sub} [mL/min] the HDF substitution rate, Q_{UF} [mL/min] the ultrafiltration rate, C_{art} [mg/dL] the plasma concentration before the dialyzer of the molecule whose clearance is to be calculated (arterial plasma), and C_{ven} [mg/dL] the plasma concentration after the dialyzer (venous plasma).

Non-clearance and non-removal related secondary variables were corrected for hematocrit (Hct) according to the following two formulas:

Blood cell counts:

$$C_t^{corr} = C_t \times Hct_{pre}/Hct_t$$

Markers of complement activation, markers of inflammation (except for blood cell counts), markers of oxidative stress, β-Thromboglobulin (β-TG), and Thromboxane (TxB2):

$$C_t^{corr} = C_t \times Hct_{pre}/Hct_t \times (100\% - Hct_t)/(100\% - Hct_{pre})$$

The corrections were calculated for each collection time point *(t)* except pre-dialysis values *(pre)*.

The primary analysis consisted of the four parts listed below. It used a gate-keeping procedure to prevent inflation of the Type 1 error rate.[12] The first two steps, which were performed in parallel, implement the Bonferroni-Holm procedure. The overall one-sided α over all tests was 2.5%.

1. Non-inferiority comparison of FX CorAL versus FX CorDiax; non-inferiority margin δ = −5%.
2. Non-inferiority comparison of FX CorAL versus xevonta; non-inferiority margin δ = −5%.
3. Superiority comparison of FX CorAL versus xevonta.
4. Superiority comparison of FX CorAL versus FX CorDiax.

Assuming no carry over effect, a linear mixed model was used for the statistical analysis. This model included the fixed effects “period“ and “dialyzer“ and the random effects “center“ and “patient“. The patients within each center identified the subjects with repeated measurements in the three periods. The comparison of the FX CorAL with each of the other two comparator dialyzers was done by defining contrasts or estimators of the fixed effect “dialyzer“.

The non-inferiority tests were based on the per protocol (PP) population which included all subjects who entered the study in accordance with each inclusion and exclusion criterion and who finished the study in accordance with the study protocol, without any major protocol deviations occurring. For validation purposes, this analysis was repeated on the intention to treat (ITT) population which included all patients who were randomized and for whom primary outcome data was available for at least one dialyzer. The superiority tests were performed on the ITT population and for validation on the PP population.

Analyses of secondary performance endpoints and PROs applied the same linear mixed model as described for the primary endpoint to calculate mean differences between FX CorAL and its comparators as well as the corresponding 95% confidence intervals.

Hemocompatibility markers were analyzed descriptively and compared between dialyzers, based on their concentration or activity profile over one HDF session. The analyses included *intra*dialytic changes vs. the baseline (pre-)value at the *session's* start. In addition, the eMPORA III study determined *inter*dialytic changes: these are changes of the pre-values of the marker within one treatment *period*, which consisted of 12 sessions with one type of dialyzer.

Blood cell counts as well as hemocompatibility and activity markers were corrected for Hct changes over an HDF session. Missing values for all safety and efficacy endpoints were not replaced and described as a distinct category in tables. Based on the ‘missing at random assumption’, the linear mixed model allowed the modelling of incomplete data.

References to Supplement

1. Buysse DJ, Reynolds CF, Monk TH, Berman SR, Kupfer DJ. The Pittsburgh Sleep Quality Index: a new instrument for psychiatric practice and research. *Psychiatry Res*. 1989;28(2):193-213. doi:10.1016/0165-1781(89)90047-4

2. Hays RD, Kallich J, Mapes D, et al. Kidney Disease Quality of Life Short Form (KDQOL-SF TM), Version 1.3: A Manual for Use and Scoring. RAND Corporation; 1997. Accessed May 16, 2023. https://www.rand.org/pubs/papers/P7994.html

3. Phan NQ, Blome C, Fritz F, et al. Assessment of pruritus intensity: prospective study on validity and reliability of the visual analogue scale, numerical rating scale and verbal rating scale in 471 patients with chronic pruritus. *Acta Derm Venereol*. 2012;92(5):502-507. doi:10.2340/00015555-1246

4. Storck M, Sandmann S, Bruland P, et al. Pruritus Intensity Scales across Europe: a prospective validation study. *J Eur Acad Dermatol Venereol JEADV*. 2021;35(5):1176-1185. doi:10.1111/jdv.17111

5. Verweyen E, Ständer S, Kreitz K, et al. Validation of a Comprehensive Set of Pruritus Assessment Instruments: The Chronic Pruritus Tools Questionnaire PRURITOOLS. *Acta Derm Venereol*. 2019;99(7):657-663. doi:10.2340/00015555-3158

6. Yosipovitch G, Reaney M, Mastey V, et al. Peak Pruritus Numerical Rating Scale: psychometric validation and responder definition for assessing itch in moderate-to-severe atopic dermatitis. *Br J Dermatol*. 2019;181(4):761-769. doi:10.1111/bjd.17744

7. Walters AS, LeBrocq C, Dhar A, et al. Validation of the International Restless Legs Syndrome Study Group rating scale for restless legs syndrome. *Sleep Med*. 2003;4(2):121-132. doi:10.1016/s1389-9457(02)00258-7

8. Wunderlich GR, Evans KR, Sills T, et al. An item response analysis of the international restless legs syndrome study group rating scale for restless legs syndrome. *Sleep Med*. 2005;6(2):131-139. doi:10.1016/j.sleep.2004.10.010

9. Abetz L, Arbuckle R, Allen RP, et al. The reliability, validity and responsiveness of the International Restless Legs Syndrome Study Group rating scale and subscales in a clinical-trial setting. *Sleep Med*. 2006;7(4):340-349. doi:10.1016/j.sleep.2005.12.011

10. Maduell F, Arias-Guillen M, Fontseré N, et al. Elimination of large uremic toxins by a dialyzer specifically designed for high-volume convective therapies. *Blood Purif*. 2014;37(2):125-130. doi:10.1159/000358214

11. Gotch FA, Panlilio FM, Buyaki RA, Wang EX, Folden TI, Levin NW. Mechanisms determining the ratio of conductivity clearance to urea clearance. *Kidney Int Suppl*. 2004;(89):S3-S24. doi:10.1111/j.1523-1755.2004.00759.x

12. Bretz F, Maurer W, Brannath W, Posch M. A graphical approach to sequentially rejective multiple test procedures. *Stat Med*. 2009;28(4):586-604. doi:10.1002/sim.3495
